# Supplementary material for: Integrating taxonomic, functional, and strain-level profiling of diverse microbial communities with bioBakery 3
Source: eLife. 2021 May 4;10:e65088. doi: 10.7554/eLife.65088 (PMC8096432; doi:10.7554/eLife.65088)
Supplement: Supplementary file 1. [file elife-65088-supp1.docx]

**Supplementary File 1: Average values of F1 scores of MetaPhlAn 3, MetaPhlAn2, mOTUs2, and Kraken species-level profiles computed on the 123 synthetic metagenomes.**

| **Tool** | **Airways** | **Gastrointestinal tract** | **Oral** | **Skin** | **Urogenital tract** | **Mouse gut** | **Non-human** |
| --- | --- | --- | --- | --- | --- | --- | --- |
| **MetaPhlAn v3.0 stat_q 0.2** | 0.880 | 0.894 | 0.869 | 0.853 | 0.869 | 0.722 | 0.830 |
| **MetaPhlAn v3.0 stat_q 0.1** | 0.896 | 0.908 | 0.898 | 0.883 | 0.906 | 0.728 | 0.855 |
| **MetaPhlAn2 v2.7** | 0.723 | 0.761 | 0.672 | 0.751 | 0.701 | 0.544 | 0.330 |
| **mOTUs251_precision** | 0.768 | 0.824 | 0.787 | 0.761 | 0.779 | 0.770 | 0.632 |
| **mOTUs251_recall** | 0.683 | 0.768 | 0.772 | 0.720 | 0.727 | 0.800 | 0.529 |
| **Bracken_208_25_refseq** | 0.440 | 0.426 | 0.525 | 0.359 | 0.339 | 0.091 | 0.021 |
