# Supplementary material for: Integrating taxonomic, functional, and strain-level profiling of diverse microbial communities with bioBakery 3
Source: eLife. 2021 May 4;10:e65088. doi: 10.7554/eLife.65088 (PMC8096432; doi:10.7554/eLife.65088)
Supplement: Supplementary file 4. [file elife-65088-supp4.docx]

**Supplementary File 4: Comparison of runtime and memory consumption of MetaPhlAn 3, MetaPhlAn2, mOTUs2, and Kraken+Bracken on the 5 HMP metagenomes.**

| **Tool** | **Elapsed time**  **(mean h)** | **Elapsed time**  **(sd h)** | **Memory Peak (mean Gb)** | **Memory Peak (sd Gb)** | **Reads per second (mean)** | **Reads per second (sd)** |
| --- | --- | --- | --- | --- | --- | --- |
| **MetaPhlAn v3.0** | 3.1120 | 2.65 | 2.614 | 0.01 | 10031.2 | 3,560.25 |
| **MetaPhlAn2 v2.7** | 8.2183 | 3.58 | 2.079 | 0.27 | 2911.2 | 400.08 |
| **mOTUs251_precision** | 10.6094 | 4.92 | 4.034 | 1.30 | 2283 | 234.26 |
| **mOTUs251_recall** | 11.5189 | 6.18 | 4.035 | 1.30 | 2186 | 310.86 |
| **Bracken_208_25_refseq** | 2.3504 | 1.40 | 32.529 | 0.27 | 11163 | 2,759.93 |
